# Insecticidal activities of *Streptomyces* sp. KSF103 ethyl acetate extract against medically important mosquitoes and non-target organisms

**DOI:** 10.1038/s41598-022-25387-9

**Published:** 2023-01-02

**Authors:** Zheng Hua Amelia-Yap, Van Lun Low, Atiporn Saeung, Fong Lee Ng, Chee Dhang Chen, Pouya Hassandarvish, Geok Yuan Annie Tan, Sazaly AbuBakar, Adzzie Shazleen Azman

**Affiliations:** 1 Higher Institution Centre of Excellence (HICoE), Tropical Infectious Diseases Research and Education Centre (TIDREC), Universiti Malaya, 50603 Kuala Lumpur, Malaysia; 2 Center of Insect Vector Study, Department of Parasitology, Faculty of Medicine, Chiang Mai University, Chiang Mai, Thailand; 3 Institute of Ocean and Earth Sciences (IOES), Universiti Malaya, 50603 Kuala Lumpur, Malaysia; 4 Institute of Biological Sciences, Faculty of Science, Universiti Malaya, 50603 Kuala Lumpur, Malaysia; 5 School of Science, Monash University Malaysia, Jalan Lagoon Selatan, 47500 Bandar Sunway, Malaysia

**Keywords:** Microbiology, Zoology

## Abstract

A potentially novel actinobacterium isolated from forest soil, *Streptomyces* sp. KSF103 was evaluated for its insecticidal effect against several mosquito species namely *Aedes aegypti*, *Aedes albopictus*, *Anopheles cracens* and *Culex quinquefasciatus*. Mosquito larvae and adults were exposed to various concentrations of the ethyl acetate (EA) extract for 24 h. Considerable mortality was evident after the EA extract treatment for all four important vector mosquitoes. Larvicidal activity of the EA extract resulted in LC_50_ at 0.045 mg/mL and LC_90_ at 0.080 mg/mL for *Ae. aegypti*; LC_50_ at 0.060 mg/mL and LC_90_ at 0.247 mg/mL for *Ae. albopictus*; LC_50_ at 2.141 mg/mL and LC_90_ at 6.345 mg/mL for *An. cracens*; and LC_50_ at 0.272 mg/mL and LC_90_ at 0.980 mg/mL for *Cx. quinquefasciatus*. In adulticidal tests, the EA extract was the most toxic to *Ae. albopictus* adults (LD_50_ = 2.445 mg/mL; LD_90_ = 20.004 mg/mL), followed by *An. cracens* (LD_50_ = 5.121 mg/mL; LD_90_ = 147.854 mg/mL) and then *Ae. aegypti* (LD_50_ = 28.873 mg/mL; LD_90_ = 274.823 mg/mL). Additionally, the EA extract exhibited ovicidal activity against *Ae. aegypti* (LC_50_ = 0.715 mg/mL; LC_90_ = 6.956 mg/mL), *Ae. albopictus* (LC_50_ = 0.715 mg/mL; LC_90_ = 6.956 mg/mL), and *An. cracens* (LC_50_ = 0.715 mg/mL; LC_90_ = 6.956 mg/mL), evaluated up to 168 h post-treatment. It displayed no toxicity on the freshwater microalga *Chlorella* sp. Beijerinck UMACC 313, marine microalga *Chlorella* sp. Beijerinck UMACC 258 and the ant *Odontoponera denticulata*. In conclusion, the EA extract showed promising larvicidal, adulticidal and ovicidal activity against *Ae. aegypti*, *Ae. albopictus*, *An. cracens*, and *Cx. quinquefasciatus* (larvae only). The results suggest that the EA extract of *Streptomyces* sp. KSF103 has the potential to be used as an environmental-friendly approach in mosquito control. The current study would serve as an initial step toward complementing microbe-based bioinsecticides for synthetic insecticides against medically important mosquitoes.

## Introduction

*Aedes*, *Anopheles* and *Culex* are prolific vectors of various mosquito-borne diseases, including dengue fever, Zika, Chikungunya, yellow fever, malaria and filariasis. Mosquitoes are becoming more prevalent and expanding their range due to climate change, socioeconomic conditions, and the ease of world travel today, triggering the rampant spread of mosquito-borne diseases worldwide^1^. Globally, mosquito-borne diseases are menacing more than four billion people in over a hundred countries. Because of their anthropophilic and host-seeking behaviour, along with the fact that they breed preferentially in artificial habitats within or near residential areas (particularly *Aedes* and *Culex*), mosquitoes are competent and epidemiologically significant species.

Suppressing mosquitoes is incredibly challenging, as they adapt well to a variety of natural and artificial habitats and quickly recover following disturbances caused by natural events or anthropogenic stresses^2^. Additionally, rigorous use of synthetic biocides and the limited chemical classes available resulted in the accumulation of toxic residues and the evolution of mosquitoes as adaptive responses to insecticide resistance. Insects' short life cycles and the abundance of offspring foster the development of populations with diverse genetic advantages, priming insect populations for insecticide resistance and impairing the efficacy of current mosquito tactics^3^. Routine insecticide application induces insecticide resistance to develop, putting many insecticide-exposed populations —particularly the population that survived the exposure—under selective pressure^4^. The current crisis necessitates the development of additional mosquito-specific insecticides that are environmentally benign, biodegradable, and cost-effective. Although biological control via natural parasites, predatory insects or fishes is crucial for establishing an environmental-oriented and integrated pest management approach, it is less effective due to the challenges surfaced from mass multiplication^5^. *Bacillus thuringiensis* var *israelensis* (*Bti*) is comparatively less effective in organically enriched water, thus may not be efficient in controlling *Culex* mosquitoes^6^. *Wolbachia*-infected mosquito dissemination, though effective in controlling disease spread, the long-term efficacy and public acceptance of *Wolbachia* remain under discussion^7^. However, the high efficacy of *Bti* against other dipteran larvae provided a choice to integrate in mosquito control programs as a potent biolarvicide should be recognized.

Given the drawbacks of synthetic insecticides and the pressing need to prevent mosquito proliferation, there has been a renaissance of interest in uncovering additional sources of biologically active compounds with negligible adverse health and environmental repercussions. Naturally occurring compounds derived from entomopathogenic microorganisms are gaining global popularity due to their relative environmental friendliness, target specificity, and cost-effectiveness^8^. In recent years, bacterial secondary metabolites (SMs) have been proposed as a source of insecticides that could be alternatives to enlarge the armoury of insecticidal compounds available^9^. The use of *Streptomyces* SMs in particular, swiftly gains public acceptance because it poses hardly any hazard to ecosystems and has little impact on humans and other non-target organisms. In contrast to conventional insecticides, some bacterial SMs consist of blends of chemical compounds which can act on a variety of novel target sites, thereby minimizing the likelihood of resistance development. Among the biological control agents derived from various entomopathogenic microorganisms, actinobacteria, notably those of the genus *Streptomyces*, represent a sizable microbial reservoir capable of producing novel bioactive compounds employed as insecticides. They synthesize a diverse array of SMs that are economically important in medicine^10,11^, with several of them exerting high toxicity against agricultural insect pests^12–15^. Certain *Streptomyces* metabolites, such as avermectin, dianemycin, milbemycin, and nanchangmycin, have been developed as protective agents against a varied spectrum of insect pests^16,17^.

The discovery that *Streptomyces* spp. produces a diverse array of SMs paves the way for these chemicals to become even more targeted and potent biological control agents for mosquitoes compared to the other commercial products. Concurrent benefits of mass production of *Streptomyces* SMs in laboratories and industry support the bioactive compounds' potential as alternative sources of mosquitocides. As evidenced by an increasing corpus of published data, *Streptomyces* SMs exhibit significant ovicidal, larvicidal, and pupacidal bioefficiency against anopheline and culicine mosquitoes^9^. While previous research has shed light on the bioactivities of *Streptomyces* SMs, there is still a dearth of evidence about their adulticidal effect. In light of the World Health Organisation (WHO)’s urgency for the development and adoption of novel control agents in the context of integrated mosquito control concerning the Global Vector Control Response 2017–2030, and motivated by *Streptomyces'* outstanding biotechnological potential as a sustainable alternative to mosquito control, the current work sought to establish a more environmentally friendly and safer alternative using *Streptomyces* sp. KSF103. Therefore, the present study aims to identify the actinobacterium using molecular characterization, isolate insecticidal extract from *Streptomyces* sp. KSF103, and evaluate the larvicidal, adulticidal, and ovicidal spectrums of the extract against *Ae. aegypti*, *Ae. albopictus*, *An. cracens*, and *Cx. quinquefasciatus*. To address the growing demand for effective and safe mosquito control agents, the toxicity of *Streptomyces* sp. KSF103 towards non-target organisms: the microalgae and ants were determined. Our results showed that members of the genus *Streptomyces* can be a valuable source of novel mosquitocides and laid the framework for future research into the use of *Streptomyces*-derived SMs as pesticides against other medically important pests.

## Materials and methods

### Isolation and characterisation of *Streptomyces* sp. KSF103

*Streptomyces* sp. KSF103 (Kuala Sat Forest number 103) was isolated from bulk soils collected from a primary forest in Jerantut, Pahang, Malaysia. Morphological characterisation of the strain was carried out according to Goodfellow and Cross (1984)^18^. The formation of aerial and substrate mycelia, and the arrangement of spores were observed under a light microscope. The strain was then purified using the streak method on yeast-malt extract agar medium (ISP Medium No. 2, ISP2) and also maintained at − 80 °C in glycerol suspension (30%, v/v).

### Taxonomic identification of isolated actinobacterium

#### Molecular characteristics of *Streptomyces* sp. KSF103

At the stationary phase, *Streptomyces* sp. KSF103 was harvested by centrifugation at 2600 rcf for ten minutes. Following that, the pellet was subsequently collected for DNA isolation. The genomic DNA was isolated using the Quick DNA Fungal/Bacterial Kit (Zymo Research, USA) according to the manufacturer's protocol. The amplification of 16S rRNA was performed using the universal primers 27F (5'-AGAGTTTGATCCTGGCTCAG-3') and 1492R (5'-TACGGCTACCTTGTTACGACTT-3'). Taq polymerase was used to amplify the 16S rRNA gene using exTEN 2X PCR Master Mix (1st Base, Singapore). The amplification reaction was performed with the extracted genomic DNA as a template following the conditions: initial denaturation at 94 °C for 4 min, 35 cycles of amplification (denaturation at 94 °C for 1 min, annealing at 54 °C for 1 min, extension at 72 °C for 1 min), and a final extension at 72 °C for 10 min. The PCR product was purified using NucleoSpin Gel and PCR Clean-up (Macherey–Nagel, Germany) as per the manufacturer's protocol.

The forward and reverse 16S rRNA gene sequences were assembled and analysed using the BioEdit Sequence Alignment Editor^19^ and compared to those of the type strains available on the EzBioCloud database^20^. Multiple alignments of the sequences were conducted using the CLUSTAL-W tool in MEGA-X. The phylogenetic tree was constructed using the MEGA-X software^21^, employing the neighbour-joining method^22^. Kimura's two-parameter model was used to calculate evolutionary distances^23^. The topology of the resultant neighbour-joining trees was evaluated by bootstrap analysis after 1000 replications^24^.

#### Physiological characteristics of *Streptomyces* sp. KSF103

The formation of aerial and substrate mycelium, and arrangement of the spore were observed under a light microscope. Physiological characterizations, including growth temperature (20–50 °C), salt concentration (0–10%) and pH (4–10), were performed.

### Fermentation and extraction of bioactive compound

The fermentation and extraction of bioactive compounds from *Streptomyces* sp. KSF103 in this study was performed following the methods of Ganesan, et al.^25^ and Thenmozhi, et al.^26^ with suitable modifications. *Streptomyces* sp. KSF103 was cultivated on yeast-malt extract (ISP2) agar plates for five days at 28 °C. Chunks of the corresponding agar with bacterial colonies were added to two 50 mL centrifuge tubes containing 30 mL of ISP2 medium. After three days of incubation at 28 °C with agitation at 120 rpm, the seed cultures were used to inoculate nine 1000 mL flasks containing 800 mL of ISP2 broth media. A total of 7.2 L of *Streptomyces* sp. KSF103 was fermented at 28 °C for 14 days at 120 rpm agitation. The resulting culture broth was separated from the mycelium by centrifugation at 2600 rcf for 30 min at 4 °C. The culture filtrates were collected and subjected to SMs extraction with ethyl acetate, an organic solvent with semi-polarity. For 4–6 days, the culture filtrates were soaked in ethyl acetate. The aqueous organic phase was separated and subsequently concentrated at 40 °C with a vacuum rotary evaporator until the eluant was colourless. The supernatant was extracted with an equal volume of ethyl acetate, and the resulting organic layers were evaporated to get the ethyl acetate extract (EA extract). The resulting EA extract was stored at − 20 °C in a sterile vial. Prior to each bioassay, stock solutions were made fresh. In the current study, two equivalent batches of EA extract tested, previously submitted to quality control, were used.

### Mosquito colonization and maintenance

Both *Ae. aegypti* and *Ae. albopictus* were long-established laboratory colonies that were reared under controlled conditions (26 ± 1 °C, 70 ± 5% relative humidity (RH) with 12 h day/night cycle) and were free from the exposures of insecticides, repellents, and pathogens for over 90 and 70 generations, respectively. The rearing method was adopted from Amelia-Yap et al.^27^. The same larval densities, diets and environmental conditions were maintained for both of these strains, provided with ground beef liver powder and yeast ad libitum. After the formation of pupae, they were transferred daily from larval trays to cups containing dechlorinated water and introduced into the rearing cage [30 cm (L) × 30 cm (W) × 30 cm (H)] where adults emerged and mated freely. Adult mosquitoes were maintained on a ten percent w/v sugar solution supplemented with vitamin B complex and blood-fed twice per week with human blood from the group of researchers (tested negative for several mosquito-borne diseases) drawn in EDTA tube using Hemotek® membrane-feeding system to promote egg production.

*Anopheles cracens* has been incriminated as the main vector of *Plasmodium knowlesi* which is the ethiological agent of human *knowlesi* malaria in Southeast Asia, with more than 1000 sporozoites in positive mosquitoes and this revealed that they are efficient vectors^28^. A susceptible laboratory reference strain that has been surviving well in the insectary was used for all the laboratory-based experiments in this study. The eggs of the *An. cracens* self-mating colony were obtained from the Department of Parasitology laboratory, Chiang Mai University, Thailand. The mosquito colony was maintained as described by Choochote and Saeung^29^ in an insectarium at a temperature of 26 °C with a RH 80 ± 5% and an alternation of 12 h day/night cycle. A few female mosquitoes were isolated in a cup and fed with human blood drawn in EDTA tube using Hemotek® membrane-feeding system to promote egg production.

The laboratory reference strain of *Culex quinquefasciatus* was maintained in the Insectary unit of Arthropod Research Laboratory, TIDREC, Universiti Malaya, which has been cultured under insecticide-free conditions for 75 generations (26 ± 1 °C, 70 ± 5% RH with 12 h day/night cycle). Freshly laid egg rafts were incubated in distilled water to ensure complete hatching. The hatched-out larvae were reared following standard techniques in plastic trays (30 cm × 25 cm × 5 cm), at the rate of 100 larvae/tray of distilled water. A pinch of finely ground TetraBits Complete® was provided to the larvae every other day. However, the mosquito colony collapsed before carrying out the ovicidal and adulticidal tests.

### Larvicidal bioassays

The test concentrations were chosen following preliminary experiments against early fourth instar larvae at a range of dosages ranging from 0.010 to 10 mg/mL. The WHO protocol for evaluating larvicidal bioassays was adopted with suitable modifications^30^. The EA extract's acute toxicity was determined by measuring mortality after 24 h of exposure in early fourth instar larvae of *Ae. aegypti*, *Ae. albopictus*, *An. cracens* and *Cx. quinquefasciatus*. Using a wide-bore plastic transfer pipette, batches of twenty-five larvae were transferred into disposable test containers. Excess water was gently removed, followed by the addition of 100 mL of dechlorinated water. To prepare the test solutions, the EA extract was diluted in dimethyl sulfoxide (DMSO) to a 200 mg/mL concentration after weighing. The appropriate volumes of dilutions were added into the disposable test containers (350 mL) to obtain the desired target concentrations, beginning with the lowest concentration. Based on the preliminary screening results, the EA extract of *Streptomyces* sp. KFS103 were subjected to concentration–response bioassay for larvicidal activity against the larvae again. The LC_50_ and LC_90_ values were determined using a restricted range of five concentrations yielding 10–95% mortality rate. Since all bioassays could not be performed concurrently, treatments were blocked across time, with a separate 1% DMSO control treatment added in each block. Each block of the bioassay was performed using freshly prepared extract solutions.

To ensure a homogeneous mixture of the chemicals, the solutions were resuspended using a micropipette and the cup was gently swirled. All bioassays were conducted under the same environmental conditions to guarantee the reproducibility between experiments. For each assay, five concentrations were tested in triplicates, and each assay was repeated three times. The mortality data was recorded 24 h post-treatment. No food was offered to the larvae. Larvae were graded as "dead" if they did not respond to tapping or prodding with a sterile micropipette tip. Larval mortality was calculated for each concentration. Mortality was converted into percent mortality. Each concentration's corrected percentage mortality value was subjected to subsequent data analysis.

### Adult topical bioassays

All experimental assays were conducted on non-blood-fed adult *Ae. aegypti*, *Ae. albopictus*, and *An. cracens* females aged four to five days, following previously described procedures of direct topical application^31^ with slight modifications to precisely determine the toxicity of the EA extract to mosquitoes. Direct topical application also permits accurate plotting of a dose–response curve to assess lethal dose (LD_50_ and LD_90_) values, the statistically derived dose required to score 50% and 90% mortality rates. Briefly, the EA extract was dissolved to a concentration of 200 mg/mL in acetone with vigorous vortexing. Different ranges of concentrations of the solution for the tests were created by further diluting the 200 mg/mL stock in acetone. The mosquitoes were first aspirated into separate clear cups. After purging the glass syringe with acetone, it was filled with the diluted EA extract and secured in the microapplicator (Hamilton PB600-1 Repeating Syringe Dispensers) that delivered a volume of 0.2 μL. Adult female mosquitoes were cold-anaesthetized in batches of five and were then placed on ice in a Petri dish. A single female was taken from the cup with a fine tweezer, and 0.2 μL of the test solution was administered to the dorsal thorax using the syringe microapplicator. To confirm the delivery of the EA extract and ensure all mosquitoes receive a given volume of the test solution, each mosquito was observed using a dissecting microscope. Cohorts of five mosquitoes were treated with a specific compound until the amount reached twenty-five females per concentration in each assay.

Subsequent dose–response assays were conducted based on screening mortality that produced a 10–95% range in initial screening to calculate LD_50_ and LD_90_. Involving five concentrations of the EA extract, twenty-five adult female mosquitoes were dosed per concentration. Females treated with 0.2 μL of acetone alone were used as the negative control. After dosing, cups were covered with a screen mesh and secured with a rubber band. Cotton balls saturated with ten percent sucrose solution were provided for each cup. All the mosquitoes were kept at 26 ± 1 °C, 70 ± 5% RH with 12 h day/night cycle during the 24-h recovery period. Mortality was recorded at 24 h post-exposure. Mosquitoes that appeared dead or lacked movements with no flying ability were scored as "dead". For each assay, five concentrations were tested in triplicates, and each assay was repeated three times.

### Ovicidal bioassays

The protocol for ovicidal bioassays was adopted from Prajapati et al.^32^ with slight modifications. To facilitate embryonation of the freshly laid eggs, the oviposition papers were stored in rearing conditions for 48 h of the drying period. Eggs were examined for any deformities following drying, and then groups of twenty-five eggs were treated separately with the extracts at 0.250, 0.500, 1, 2, 4 mg/mL concentrations in a six-well plate. Eggs treated with 1% of DMSO in water were used as the negative control groups. For each assay, five concentrations were tested in triplicates, and each assay was repeated three times. The ovicidal activity was evaluated up to 168 h post-treatment and subsequently, both treated and negative control eggs were observed under the microscope. The non-hatched eggs with unopened opercula were counted in each treatment. The percent mortality was calculated using the following formula:

$${\text{Percentage\,\, of \,\,egg \,\,mortality}}= \frac{{\text{No.\,\, of\,\, unhatched eggs}}}{{\text{Total \,\, no \,\,of\,\, eggs \,\,introduced}}} \times 100\%$$

### Environmental toxicity to non-target organisms

The environmental toxicity of *Streptomyces* sp. KSF103 EA extract was determined against microalgae and ants. Microalgae, including *Chlorella* spp., are often used as bioindicators in detecting contaminants as they are sensitive to contaminations^33^. Ants, including *Odontoponera* spp., can be chronically exposed to insecticide residues after prolonged applications. This is of great concern because ants play multiple crucial roles for ecosystem functioning^34^. The current study involved the freshwater microalga *Chlorella* sp. Beijerinck UMACC 313 and the marine microalga *Chlorella* sp. Beijerinck UMACC 258 for non-target toxicity tests. Both strains of tropical algae were obtained from the University of Malaya Algae Culture Collection (UMACC)^35^. The freshwater microalga was grown in Bold's Basal Medium, while the marine microalga was cultured in Prov Medium. An inoculum size of 20%, standardized at an optical density at of 0.2 at 620 nm (OD_620_ nm) from exponential phase cultures was used. Each microalga was grown in triplicate in a total volume of 500 mL and added to tubes containing the *Streptomyces* sp. KSF103 EA extract at the LC_90_ value (Table 2—6.956 mg/mL) or 1% DMSO (negative control) in a final reaction volume of 10 mL of the medium. At 0, 4, 8 and 12 days of incubation at 25 °C, the growths of both microalgal strains were monitored based on OD_620_ nm, adopted from Ng et al.^36^. Other biochemical profiling and photosynthetic performance of the microalgae such as chlorophyll-a (chl-a), carotenoid, maximum quantum yield (Fv/Fm), alpha, maximum relative electron transport rate (rETRmax) and photoadaptive index (Ek) were measured.

Biomass of algal cultures were estimated based on chl-*a* and carotenoid content. The chl-*a* and carotenoid concentrations were determined using the spectrophotometric method^37^. Fluorescence analysis was employed to measure the photosynthetic performance of algal cultures in both *Streptomyces* sp. KSF103 EA extract and DMSO treatment. Photosynthetic parameters in this study were measured using a pulse amplitude modulation (PAM) fluorometer (Diving PAM, Walz, Germany)^38,39^.

The effect of *Streptomyces* sp. KSF103 EA extract was also evaluated on *Odontoponera denticulata*, a species commonly found in Malaysia. Adults female *O. denticulata* were obtained from the wild. They were exposed to LD_90_ concentration (Table 3—274.823 mg/mL) of the extract with acetone. Similar to adult topical bioassays, 0.2 μL of the test solution was administered to the dorsal thorax of the ants using the syringe microapplicator. They were then (20 adults) transferred to a rearing cage with rearing conditions of 26 ± 1 °C, 70 ± 5% RH with 12 h day/night cycle. Acetone was used as a negative control. Mortality was determined after 4 days.

### Data analyses

Mosquito mortality in the control groups that ranged from 0 to 10% should be corrected accordingly using Abbott's formula^40^. Dataset with mortality rate greater than 10% were not considered. The average larval and egg mortality data were subjected to a probit regression analysis to calculate the lethal concentrations LC_50_ and LC_90_ using SPSS (version 26). In the case of adulticidal testing, LD_50_ and LD_90_ values were calculated. Other statistics at 95% confidence limits of upper confidence limits (UCL) and lower confidence limits (LCL), degree of freedom (df), and chi-square values were also calculated^41^. Moreover, GraphPad Prism version 9 (GraphPad Software, La Jolla, CA, USA) was used to analyze dose–response mortality data in best-fit sigmoidal plots with the minimum and maximum constrained to 0 and 100%, respectively. Using the same software, the effect of the EA extract treatment was determined by a one-way analysis of variance (ANOVA). If the analysis of variance showed significant differences between the groups (P ≤ 0.05), a Dunnett's post-hoc test (P ≤ 0.05) was performed to determine the significant difference between control and multiple treatment groups.

The environmental toxicity of the *Streptomyces* sp. KSF103 against *Chlorella* sp. Beijerinck UMACC 313 and *Chlorella* sp. Beijerinck UMACC 258 was assessed by reading the optical density at 620 nm, and the results were compared with the negative control using a two-way ANOVA from the GraphPad Prism software combined with the Bonferroni test (P < 0.05). All experimental data were expressed as the mean ± standard deviation and analyzed at 5% of significance level (P < 0.05).

## Results

### 16S rRNA gene sequence and phylogenetic analysis

**Figure 1:**
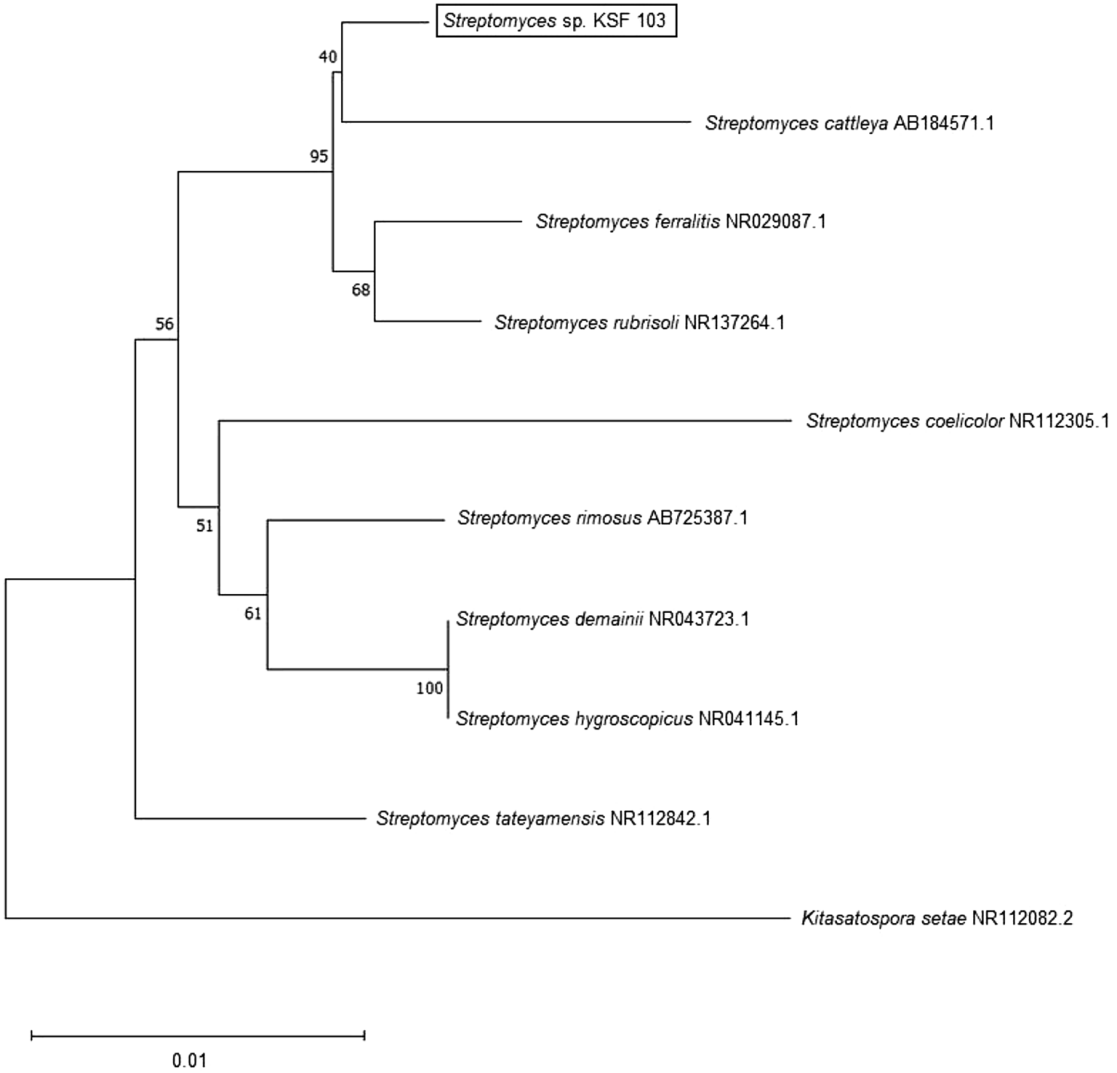
Phylogenetic tree based on the 16S rRNA gene sequences using the neighbour-joining method for the *Streptomyces* sp. KSF103 and its closely related type strains. The number of branch nodes is bootstrap values (1000 resampling). The scale bar represents 0.01 substitutions per site.

The almost complete 16S rRNA gene sequence for strain KSF103 (1336 bp) was deposited in the GenBank under accession number MT355788. The identification of the 16S rRNA gene sequence using the EzBioCloud database showed that strain KSF103 has the highest sequence similarity to strain *Streptomyces rubrisoli* FXJ1.725(T) (99.18%), *Streptomyces ferralitis* SFOp68(T) (98.88%), *Streptomyces rimosus* subsp. *rimosus* ATCC 10,970(T) (98.43%), *Streptomyces cattleya* NRRL 8057(T) (98.43%) and *Streptomyces tateyamensis* DSM41969(T) (98.35%). From the phylogenetic tree (Fig. 1) constructed based on the 16S rRNA gene sequences employing the neighbour-joining method, it presented that strain KSF103 formed a distinct clade with type strains *S. rubrisoli* FXJ1.725(T) and *S. ferralitis* SFOp68(T) at a bootstrap value of 40%.

### Physiological characteristics of *Streptomyces* sp. KSF103

Earlier, the strain was identified based on its basic morphology on ISP2 media^18^. *Streptomyces* sp. KSF103 demonstrated an ingrowing shrivelled morphology, aerial hyphae, and a well-developed filamentous mycelium with spores. Thus, *Streptomyces* sp. KSF103 was distinguished as a member of the genus *Streptomyces*. *Streptomyces* sp. KSF103 is a Gram-positive aerobic bacterium. It grew at temperatures between 20 and 40 °C (optimum at 30–35 °C) and on a medium with a pH of 5–8 (optimum at pH 6–7). A study of *Streptomyces* sp. KSF103's tolerance to NaCl revealed that it could grow in media containing less than 2% NaCl.

### Larvicidal activity and determination of lethal concentrations

**Figure 2:**
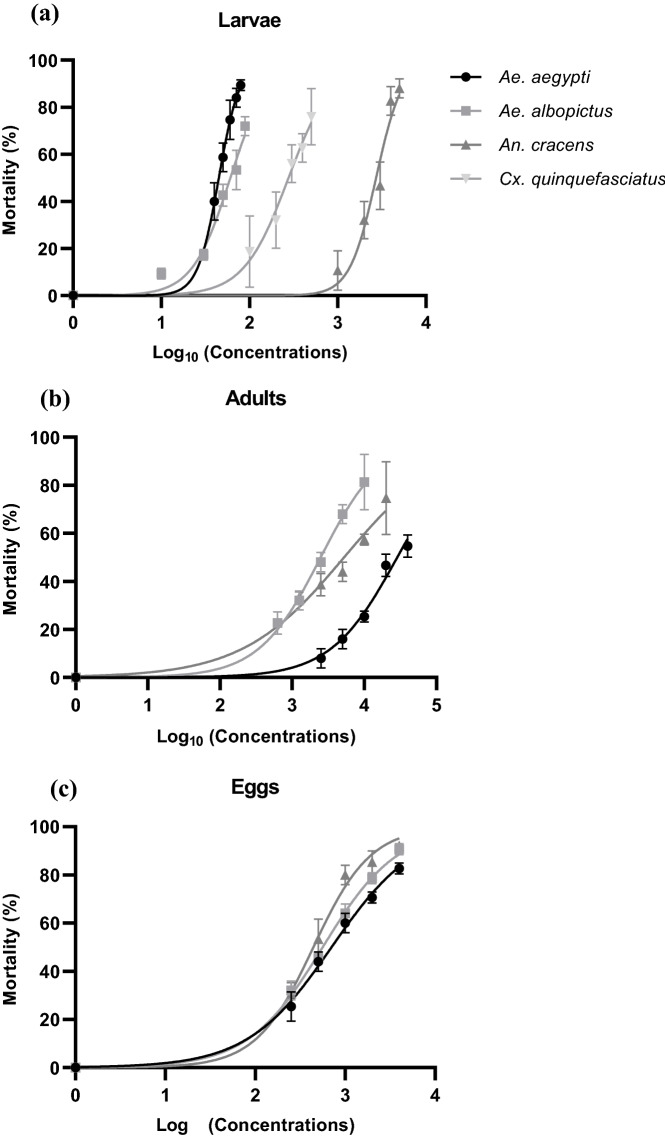
Concentration- and dose–response curves generated from the mean percentage mortality after 24 h exposure to various concentrations of *Streptomyces* sp. KSF103 EA extract for: (**a**) larvae of *Ae. aegypti*, *Ae. albopictus*, *An. cracens*, and *Cx. quinquefasciatus*; (**b**) female adults of *Ae. aegypti*, *Ae. albopictus*, and *An. cracens*; and (**c**) eggs of *Ae. aegypti*, *Ae. albopictus*, and *An. cracens*.

At 24 h post-treatment, the EA extract of *Streptomyces* sp. KSF103 exhibited concentration-dependent larvicidal activity against *Ae. aegypti*, *Ae. albopictus*, *An. cracens*, and *Cx. quinquefasciatus* larvae (Table 1, Fig. 2a). The final test concentrations for *Ae. aegypti* were 0.040, 0.050, 0.060, 0.070, and 0.080 mg/mL; 0.010, 0.030, 0.050, 0.070, and 0.090 mg/mL for *Ae. albopictus*; 1, 2, 3, 4, and 5 mg/mL for *An. cracens*; and 0.100, 0.200, 0.300, 0.400 and 0.500 mg/mL for *Cx. quinquefasciatus*. There was a significant increase in the mortality percentage with the increase in concentrations of *Streptomyces* sp. KSF103 EA across the different blocks of tests. The lethal concentrations displayed 50% (LC_50_) and 90% (LC_90_) larval mortality, chi-squared (χ^2^) values, and 95% confidence limits for the EA extract of *Streptomyces* sp. KSF103 were shown in Table 2 for larvae of all the species tested. The larvicidal activities varied between the mosquito species. The EA extract of *Streptomyces* sp. KSF103 exerted remarkable toxicity on *Ae. aegypti* with the lowest LC_50_ and LC_90_ values of 0.045 and 0.080 mg/mL, respectively. The larvae of *Ae. aegypti* exhibited a high death rate of 40.00% at 0.040 mg/mL and 89.33% at 0.080 mg/mL. A one-way ANOVA test found a significant effect of the EA extract treatment (P < 0.0001). A post hoc Dunnett's test discovered significant differences in mortality rate between all concentrations of the treatment groups and the DMSO-only control group. *Ae. albopictus*, on the other hand, was discovered to be more tolerable to the EA extract than *Ae. aegypti*, with LC_50_ and LC_90_ values of 0.060 and 0.247 mg/mL, respectively. Although the one-way ANOVA showed a significant difference (P < 0.0001) in *Ae. albopictus*, the EA extract was fatal to 84.00% of *Ae. aegypti* but only to 53.33% of *Ae. albopictus* at 0.070 mg/mL. A post hoc Dunnett's test displayed significant differences in mortality of all concentrations compared to the DMSO-only control, except for the lowest concentration (0.010 mg/mL).

**Table 1:** Mean mortalities of *Ae. aegypti* (larvae, adults and eggs), *Ae. albopictus* (larvae, adults and eggs), *An, cracens* (larvae, adults and eggs), and *Cx. quinquefasciatus* (larvae only) on different concentrations of *Streptomyces* sp. KSF103 EA extract.

	Concentration (mg/mL)	n ± SD	% ± SD
**Larvae**			
*Ae. aegypti*	0.040	10.00 ± 2.00**	40.00 ± 8.00**
	0.050	14.67 ± 1.53**	58.67 ± 6.11**
	0.060	18.67 ± 2.08**	74.67 ± 8.33**
	0.070	21.00 ± 1.00**	84.00 ± 4.00**
	0.080	22.33 ± 0.58**	89.33 ± 2.31**
	DMSO control	0.00 ± 0.00	0.00 ± 0.00
One-way ANOVA	P-value	P < 0.0001	
	F (DFn, DFd)	F (5, 12) = 105.4	
*Ae. albopictus*	0.010	2.33 ± 0.58	9.33 ± 2.31
	0.030	4.33 ± 0.58*	17.33 ± 2.31*
	0.050	10.67 ± 1.15**	42.67 ± 4.62**
	0.070	13.33 ± 2.08**	53.33 ± 8.33**
	0.090	18.00 ± 1.00**	72.00 ± 4.00**
	DMSO control	0.00 ± 0.00	0.00 ± 0.00
One-way ANOVA	P-value	P < 0.0001	
	F (DFn, DFd)	F (5, 12) = 120.3	
*An. cracens*	1.00	2.67 ± 2.08	10.67 ± 8.33
	2.00	8.00 ± 2.00	32.00 ± 8.00*
	3.00	11.67 ± 2.52	46.67 ± 10.07**
	4.00	20.67 ± 1.53	82.67 ± 6.11**
	5.00	22.00 ± 1.00	88.00 ± 4.00**
	DMSO control	0.00 ± 0.00	0.00 ± 0.00
One-way ANOVA	P-value	P < 0.0001	
	F (DFn, DFd)	F (5, 12) = 82.83	
*Cx. quinquefasciatus*	0.10	4.67 ± 3.79	18.67 ± 15.14
	0.20	8.00 ± 3.00	32.00 ± 12.00*
	0.30	14.00 ± 2.00	56.00 ± 8.00*
	0.40	15.67 ± 1.53	62.67 ± 6.11**
	0.50	19.00 ± 3.00	76.00 ± 12.00**
	DMSO control	0.00 ± 0.00	0.00 ± 0.00
One-way ANOVA	P-value	P < 0.0001	
	F (DFn, DFd)	F (5, 12) = 24.32	
**Adults**			
*Ae. aegypti*	2.50	2.00 ± 1.00	8.00 ± 4.00
	5.00	4.00 ± 1.00*	16.00 ± 4.00*
	10.00	6.33 ± 0.58**	25.33 ± 2.31*
	20.00	11.67 ± 1.15**	46.67 ± 4.62*
	40.00	13.67 ± 1.15**	54.67 ± 4.62*
	Acetone control	0.00 ± 0.00	0.00 ± 0.00
One-way ANOVA	P-value	P < 0.0001	
	F (DFn, DFd)	F (5, 12) = 105.5	
*Ae. albopictus*	0.625	5.67 ± 1.15*	22.67 ± 4.62*
	1.25	8.00 ± 1.00*	32.00 ± 4.00*
	2.50	12.00 ± 1.00**	48.00 ± 4.00*
	5.00	17.00 ± 1.00**	68.00 ± 4.00*
	10	20.33 ± 2.89**	81.33 ± 11.55*
	Acetone control	0.00 ± 0.00	0.00 ± 0.00
One-way ANOVA	P-value	P < 0.0001	
	F (DFn, DFd)	F (5, 12) = 79.88	
*An. cracens*	1.25	8.33 ± 0.58	33.33 ± 2.31*
	2.50	9.67 ± 1.15	38.67 ± 4.62**
	5.00	11.00 ± 1.00	44.00 ± 4.00**
	10.00	14.33 ± 0.58	57.33 ± 2.31**
	20.00	18.67 ± 3.79	74.67 ± 15.14**
	Acetone control	0.00 ± 0.00	0.00 ± 0.00
One-way ANOVA	P-value	P < 0.0001	
	F (DFn, DFd)	F (5, 12) = 40.94	
**Eggs**			
*Ae. aegypti*	0.25	6.33 ± 1.53**	25.33 ± 6.11*
	0.50	11.00 ± 1.00**	44.00 ± 4.00*
	1.00	15.00 ± 1.00**	60.00 ± 4.00*
	2.00	17.67 ± 0.58**	70.67 ± 2.31*
	4.00	20.67 ± 0.58**	82.67 ± 2.31*
	DMSO control	0.00 ± 0.00	0.00 ± 0.00
One-way ANOVA	P-value	P < 0.0001	
	F (DFn, DFd)	F (5, 12) = 211.0	
*Ae. albopictus*	0.25	8.00 ± 1.00**	32.00 ± 4.00*
	0.50	11.33 ± 0.58**	45.33 ± 2.31*
	1.00	16.00 ± 1.00**	64.00 ± 4.00*
	2.00	19.67 ± 0.58**	78.67 ± 2.31*
	4.00	22.67 ± 0.58**	90.67 ± 2.31*
	DMSO control	0.00 ± 0.00	0.00 ± 0.00
One-way ANOVA	P-value	P < 0.0001	
	F (DFn, DFd)	F (5, 12) = 412.4	
*An. cracens*	0.25	7.67 ± 1.15	30.67 ± 4.62**
	0.50	13.33 ± 2.08	53.33 ± 8.33**
	1.00	20.00 ± 1.00	80.00 ± 4.00**
	2.00	21.33 ± 1.15	85.33 ± 4.62**
	4.00	23.00 ± 0.00	92.00 ± 0.00**
	DMSO control	0.00 ± 0.00	0.00 ± 0.00
One-way ANOVA	P-value	P < 0.0001	
	F (DFn, DFd)	F (5, 12) = 183.2	

Negative control–nil mortality, a mean value of three replicates, * significant at P < 0.05 level, ** significant at P < 0.0001 level.

**Table 2:** Lethal concentrations of *Streptomyces* sp. KSF103 EA extract on the larvae (24 h exposure) and eggs (up to 168 h post-treatment) of *Ae. aegypti*, *Ae. albopictus*, *An. cracens* and *Cx. quinquefasciatus* (larvae only) (n = 1200).

	LC_50_ (mg/mL)	95% CL	LC_90_ (mg/mL)	95% CL	χ^2^	df	Regression equation	r^2^
**Larvae**								
*Ae. aegypti*	0.045	0.040–0.048	0.08	0.073–0.094	1.1	3	y = 5.0507x + 11.809	0.9986
*Ae. albopictus*	0.060	0.039–0.123	0.247	0.121–4.469	18.806	3	y = 1.9425x + 7.373	0.9055
*An. crascens*	2.141	1.285–2.972	6.345	4.148–25.130	8.217	3	y = 3.5844x + 3.649	0.9126
*Cx. quinquefasciatus*	0.272	0.237–0.312	0.98	0.737–1.553	2.248	3	y = 2.2753 + 6.2936	0.9672
**Eggs**								
*Ae.aegypti*	0.715	0.543–0.909	6.956	4.392–14.540	0.607	3	y = 1.2972x + 5.1674	0.9948
*Ae. albopictus*	0.559	0.427–0.698	4.126	2.883–7.1	0.315	3	y = 1.4911x + 5.3826	0.9961
*An. crascens*	0.441	0.335–0.547	2.609	1.955–3.955	3.571	3	y = 1.5905x + 5.5086	0.994

*LC*_*50*_ lethal concentration that kills 50% of the exposed organisms, *LC*_*90*_ lethal concentration that kills 90% of the exposed organisms, *UCL* upper confidence limit, *LCL* lower confidence limit, *χ*^*2*^ Chi square, *df* degrees of freedom, *r*^*2*^ regression co-efficient.

The highest LC_50_ and LC_90_ values for *An. cracens* larvae were observed in the test concentrations of 2.141 mg/mL and 6.345 mg/mL at 24 h of exposure, respectively. The mortality percentage of the larvae was merely 10.67% at the lowest concentration of 1 mg/mL tested against the larvae, which this concentration was considerably high in the other three species. The EA extract also exhibited larvicidal activity in *Cx. quinquefasciatus*, showing LC_50_ value of 0.272 mg/mL and LC_90_ value of 0.980 mg/mL. A one-way ANOVA test found a significant effect of the EA extract treatment (P < 0.0001) for both *An. cracens* and *Cx. quinquefasciatus*. Moreover, a post hoc Dunnett's test displayed significant differences in mortality of all concentrations compared to the DMSO-only control, except for the lowest concentrations of 1 mg/mL for *An. cracens* and 0.100 mg/mL for *Cx. quinquefasciatus*.

In the control groups treated with 1% DMSO, larvicidal activity was absent for all the species tested. All larvae were active and exhibited normal movement, with no larvae mortality throughout the data recording period, indicating that the larvicidal activity was caused by the EA extract and had no relation to the DMSO. In treatment groups, dead larvae were observed settling down to the bottom of the test containers. Restless movement and tail nibbling were also observed in treated larvae in behavioural analysis.

### Adulticidal activity and determination of lethal doses

The average mortality rate of the adulticidal activity of *Streptomyces* sp. KSF103 EA extract against *Ae. aegypti*, *Ae. albopictus* and *An. cracens* 24 h post-exposure is shown in Table 1, exerting variable toxicological activities. The final test concentrations for *Ae. aegypti* were 2.500, 5, 10, 20 and 40 mg/mL; 0.625, 1.250, 2.500, 5, 10 mg/mL for *Ae. albopictus*; and 1.250, 2.500, 5, 10 and 20 mg/mL for *An. cracens*. The mature stage of these mosquito species was susceptible to the EA extract in a dose-dependent manner (Fig. 2b). Adults *Ae. albopictus* revealed a 22.67% mortality rate at 0.625 mg/mL and increased to 81.33% at 10 mg/mL. The lethal dose LD_50_ value of 2.445 mg/mL and LD_90_ value of 20.004 mg/mL were required to kill 50 and 90% of the adults, respectively. A one-way ANOVA test found a significant effect of the EA extract treatment (P < 0.0001). Dunnett's post hoc test revealed significant differences in mortality of all concentrations compared to that of the DMSO-only control. Adults *Ae. aegypti* were more tolerable to the EA extract since significant mortality (P < 0.05) did not occur until 5 mg/mL (Table 1), with an LD_50_ value of 28.873 mg/mL and an LD_90_ value of 274.823 mg/mL (Table 3). At a concentration of 10 mg/mL of the EA extract, the adult death rate of *Ae. albopictus* reached 81.33%. Under the same concentration, *Ae. aegypti* had a mortality rate of only 25.33%. While a significant difference was observed (P < 0.0001), *Ae. aegypti* was the most tolerant species tested against the extract. Except for the lowest concentration examined (2.500 mg/mL), all other four concentrations were associated with significant mortality in *Ae. aegypti* adults (one-way ANOVA and Dunnett's test). As detailed in Table 1, the EA extract of *Streptomyces* sp. KSF103 was also effective against the adults of *An. cracens*, showing an LC50 of 5.121 mg/mL while the LD_90_ was 147.854 mg/mL. A one-way ANOVA test found a significant effect of the EA extract treatment (P < 0.0001). A post hoc Dunnett's test discovered significant differences in mortality rate between all concentrations of the treatment groups and the DMSO-only control group. Adults from the control groups without the EA extract addition survived.

**Table 3:** Lethal doses of *Streptomyces* sp. KSF103 EA extract on the adults (24 h exposure) of *Ae. aegypti*, *Ae. albopictus*, and *An. cracens* (n = 1200).

	LD_50_ (mg/mL)	95% CL	LD_90_ (mg/mL)	95% CL	χ^2^	df	Regression equation	r^2^
**Adults**								
*Ae. aegypti*	28.873	21.633–43.903	274.823	137.611–890.598	1.127	3	y = 1.314x + 3.08	0.9848
*Ae. albopictus*	2.445	1.945–3.069	20.004	12.786–40.115	0.691	3	y = 1.4004x + 4.608	0.991
*An. cracens*	5.121	3.570–7.411	147.854	57.991–1001.205	2.549	3	y = 0.8844x + 4.3776	0.925

*LD*_*50*_ lethal dose that kills 50% of the exposed organisms, *LD*_*90*_ lethal dose that kills 90% of the exposed organisms, *UCL* upper confidence limit, *LCL* lower confidence limit, *χ*^*2*^ Chi square, *df* degrees of freedom, *r*^*2*^ regression co-efficient.

### Ovicidal activity and determination of lethal concentrations

The ovicidal activities of *Streptomyces* sp. KSF103 EA extract on the eggs of *Ae. aegypti*, *Ae. albopictus*, and *An. cracens* are summarised in Table 1. The EA extract was highly lethal to the eggs of all of the species tested in a concentration-dependent pattern (Fig. 2c). The highest mean mortality was shown by *An. cracens*, followed by *Ae. albopictus* and *Ae. aegypti* at concentrations of 0.25, 0.5, 1, 2 and 4 mg/mL. After 168 h post-treatment, the EA extract exhibited 90.67% ovicidal efficacy against *Ae. albopictus* at a concentration of 4 mg/mL. While at the lowest concentration of 0.250 mg/mL of the EA extract, 32.00% egg mortality was observed in *Ae. albopictus*. The EA extract showed a LC_50_ value of 0.559 mg/mL and LC_90_ value of 2.609 mg/mL at 168 h post-treatment. *Ae. aegypti* eggs were more tolerant to the EA extract of *Streptomyces* sp. KSF103 at all concentrations. After 168 h post-treatment, 82.67% ovicidal activity was noticed at the highest concentration of 4 mg/mL, whereas the lowest concentration of 0.250 mg/mL demonstrated 25.33% of ovicidal activity in *Ae. aegypti*. Corresponding to the mortality rate, the highest LC_50_ and LC_90_ values were recorded at 0.441 and 6.956 mg/L, respectively Ovicidal bioassays conducted on *An. cracens* eggs showed that the *Streptomyces* sp. KSF103 EA extract had the most toxic ovicidal activity, with an LC_50_ value of 0.441 mg/mL and an LC_90_ value of 2.609 mg/mL. In three of these species tested, a one-way ANOVA showed a significant difference between the groups (P < 0.0001), and a post hoc Dunnett's test revealed further statistically significant differences when each concentration was compared to the DMSO-only control (Table 1). In the DMSO-only control, the hatchability of eggs of all of the species was normal. The overall results revealed that the mortality rate increased with increased extract concentrations.

### Toxicity of *Streptomyces* sp. KSF103 EA extract against non-target organisms

Toxicity of the EA extract of *Streptomyces* sp. KSF103 was evaluated on organisms inhabit in water and land to estimate environmental toxicity. In comparison to the control groups, the EA extract did not exert any toxicity effect on *O. denticulata* until the fourth day of the treatment (P < 0.05) and on both freshwater and marine microalgal strains at 0-, 4-, 8- and 12-days post-treatment (Table 4). The EA extract significantly increased the growth rate of UMACC 313 *Chlorella* sp. at 4-, 8- and 12-days post-treatment (P < 0.05) and UMACC 258 *Chlorella* sp. at 8- and 12-days post-treatment (P < 0.05). Other biochemical profiling and photosynthetic performance of the microalgae such as chl-a, carotenoid, Fv/Fm, alpha, rETRmax and Ek were supplemented as supporting information (see Supplementary Table S1 online).

**Table 4:** Environmental toxicity of *Streptomyces* sp. KSF103 EA extract to the microalgae UMACC 313 *Chlorella* sp. and UMACC 258 *Chlorella* sp., and insect *O. denticulata.*

Compound	OD_620nm_		% Survival at day 4 *Odontoponera denticulata*
	UMACC 313 *Chlorella* sp. (Freshwater)	UMACC 258 *Chlorella* sp. (Marine)	
***Streptomyces* sp. KSF103 EA extract**			
Day 0	0.101 ± 0.002	0.104 ± 0.006	100%
Day 4	0.349 ± 0.036*	0.362 ± 0.031	100%
Day 8	0.542 ± 0.055*	0.692 ± 0.056*	N/A
Day 12	0.608 ± 0.014*	0.713 ± 0.080*	N/A
**Dimethyl sulfoxide (Acetone for *O. denticulata*)**			
Day 0	0.104 ± 0.002	0.103 ± 0.009	100%
Day 4	0.130 ± 0.013	0.281 ± 0.004	100%
Day 8	0.160 ± 0.019	0.322 ± 0.044	N/A
Day 12	0.149 ± 0.014	0.346 ± 0.015	N/A

Data expressed as mean ± standard deviation of the OD_620nm_ of both microalgal strains, and as percentage survival for *O. denticulata.*

*Significant at P < 0.05 level versus negative control.

## Discussion

The microbial world remains the largest unexplored reservoir of biodiversity on the earth. *Streptomyces* spp. are a highly diverse group of prokaryotes ubiquitous in nature, such as soil, freshwater, and marine ecosystems. Terrestrial actinobacteria are remarkable producers of an assortment of SMs with insecticidal properties^42^; however, the primary tropical rainforest representatives have received far less attention in this regard. Therefore, primary tropical rainforest soil representing Malaysia's specific biological niche was chosen to unravel novel mosquitocide in the current study area. The bulk soils were collected and then proceeded with isolation of *Streptomyces*. One of the isolated *Streptomyces* spp., tentatively named *Streptomyces* sp. KSF103, evidently displayed outstanding towards four mosquito species *Ae. aegypti*, *Ae. albopictus*, *An. cracens*, and *Cx quinquefasciatus*.

While *Streptomyces* sp. KSF103 may appear to be a potentially novel *Streptomyces*, more specialized tests, for examples serotyping and antibiotic inhibition patterns, are needed to identify this organism as a novel bacterium. The current study uncovered the mosquitocidal potential of *Streptomyces* sp. KSF103. In the present study, the result revealed that the EA extract of *Streptomyces* sp. KSF103 displayed high bio-efficacy with low LC_50_ and LC_90_ values against *Ae. aegypti*, *Ae. albopictus*, *An. cracens*, and *Cx. quinquefasciatus* larvae. The larvicidal activity of *Streptomyces* sp. KSF103 EA extract is concordant with several past studies, including the toxicity of *Streptomyces citreofluorescens* against *Anopheles stephensi*, *Cx. quinquefasciatus* and *Ae. aegypti* larvae^43^; *Streptomyces microflavus* against *Culex pipens*^44^; and *Streptomyces* sp. VITPK9 against *Anopheles subpictus*, *Culex gelidus*, and *Culex tritaeniorhynchus*^45^. Larvae of both *Aedes* spp. demonstrated similar sensitivities to the *Streptomyces* sp. KSF103 EA extract, that greatly exceeded the mortality rates shown by *Cx. quinquefasciatus* and *An. cracens*. The low LC_50_ values obtained from the *Aedes* spp. in this study are comparable with findings obtained in a previous study accomplished by Naine and Devi^46^, who reported a similar range of larval mortality concentrations (0.060–0.400 mg/mL) when using the EA extract of *Streptomyces* sp. VITJS4 for 24 h exposure against *Ae. aegypti* larvae, with LC_50_ of 112.78 ppm (≈ 0.113 mg/mL) and LC_90_ of ≈33.642 ppm (≈ 0.336 mg/mL). The EA extract of *Streptomyces vinaceusdrappus* (S12–4) also demonstrates similar LC_50_ values, ranging from 0.140 to 0.170 mg/mL for *An. stephensi* and *Cx. quinquefasciatus*^25^.

Among the four species tested, *Ae. aegypti* larvae showed the greatest susceptibility to the EA extract of *Streptomyces* sp. KSF103, followed by *Ae. albopictus*, *Cx. quinquefasciatus* and then *An. cracens*, which was the least susceptible. The varying susceptibilities may possibly be due to the differences in the physiological characteristics of these mosquito species. This finding agrees with past studies in which the EA extract of *Streptomyces* sp. VITPK9 showed a higher LC_50_ value (0.830 mg/mL) against *An. subpictus* in relative to *Cx. gelidus* (LC_50_ = 0.150 mg/mL) and *Cx. tritaeniorhynchus* (LC_50_ = 0.489 mg/mL)^45^; *Streptomyces* VITSTK7 sp. EA extract against *An. subpictus* with a higher LC_50_ value (0.220 mg/mL) compared to *Cx. quinquefasciatus* (0.195 mg/mL)^26^.

The positive larvicidal results agree with another comparable study by Shanmugasundaram and Balagurunathan^47^, who reported a more promising result using silver nanoparticles synthesized from *Streptomyces* sp. M25 against *Ae. aegypti* larvae, with LC_50_ values of 0.06023 mg/mL and 0.039664 mg/mL from AgNPS and AgNO_3_ for 24 h exposure respectively. While silver nanoparticles are substantially more toxic than the extract alone^48^, the current study's comparable LC_50_ values fortify further research into the silver nanoparticles synthesized by *Streptomyces* sp. KSF103 in order to achieve even lower lethal concentrations.

Apart from isolating pure *Streptomyces* compounds, crude extracts are being evaluated for insecticidal activity in some cases. While unrefined extracts appear to have lower insecticidal efficacy than purified chemicals, the advantages include extremely minimal risk of target insects developing resistance to such constituents because of the presence of several bioactive compounds^49–51^. Additionally, the EA extract of *Streptomyces* sp. KSF103 had a lethal concentration capable of killing 50% (LC_50_) value of less than 100 mg/mL 24 h after treatment, which is the concentration proposed by the WHO^30^ to select potential larvicides.

One of the major problems in mosquito control is that *Aedes* eggs are resistant to desiccation periods, and they remain latent until almost the end of embryonic development^52^. *Streptomyces*-derived extracts have been reported for their ovicidal effect against different species of mosquitoes. *Streptomyces enissocaesilis* S12–17 EA extract exerted 100% ovicidal mortality at 500 ppm^53^, and the LC_50_ value of *Streptomyces vinaceusdrappus* (S12–4) EA extract for *Anopheles stephensi* was 168.62 ppm and *Cx. quinquefasciatus* was 224.53 ppm. The EA extract demonstrated ovicidal properties, with efficacies varying according to the mosquito species and concentrations. The semi-permeable endochorion and exochorion layers of mosquito eggs, through which some molecules can penetrate, could explain the ovicidal property discovered in this study^52^. A serosal cuticle forms on the inner surface of the endochorion during development and shields the embryo from external factors like desiccation or the presence of bacteria or insecticides. However, this serosal cuticle can be disrupted by lipophilic substances^54^. With lipopeptide compounds are often being detected from *Streptomyces* spp. extracts from past studies, there may be a similar group of compounds contained in the EA extract used in the current study. The SMs of *Streptomyces* sp. KSF103 may intervene in egg development by inducing eggshell thickening to inhibit hatching and impeding the developing embryo's respiration, or permeating the egg's inner regions to obstruct gaseous exchange, enzymatic and hormonal functions^55^. Although the exact mechanism varies between eggs from different mosquito species, the accumulation of evidence from prior investigations proved that *Streptomyces* SMs possess the ability in egg hatching inhibition^53,56^.

Data presented in this study demonstrated that the EA extract of *Streptomyces* KSF103 elicited intense adulticidal activity against *Ae. aegypti*, *Ae. albopictus*, and *An. cracens* adult mosquitoes with low LD_50_ and LD_90_ values. Similar reports on *Streptomyces*-derived EA extract yielded significant pesticidal activity against adult *Myzus persicae* by *Streptomyces albidoflavus* G30^15^ and *Tetranychus urticae* by the purified compound of *Streptomyces microflavus* neau3^57^ under laboratory conditions. Apart from agricultural pests, the EA extract produced from *Streptomyces* sp. VITSTK7 exhibited acaricidal activity against *Haemaphysalis bispinosa* and *Rhipicephalus microplus*^26^. To the best of our knowledge, there is a dearth of mosquito adulticidal testing using *Streptomyces*-derived extract.

The EA extract of *Streptomyces* sp. KSF103 with ovicidal, larvicidal and adulticidal activities against different mosquito species tested in this study were not toxic to UMACC 313 *Chlorella* sp. (freshwater), UMACC 258 *Chlorella* sp. (marine) and *O. denticulata*. This suggests that they may be relatively safe to the environment. The phytoplanktonic organisms, which make up the first level of the food chain, are one of the first organisms to be impacted by insecticide usage. In aquatic ecosystems, algae play an important role. Planktonic algae are crucial indicators of the various effects of compounds discharged into the aquatic system^58^. Worldwide usage of insecticides has a major impact on algae’s ability to survive, develop, and reproduce. Thus, studies on the effects of insecticides on algae are critical; when insecticides are applied in areas inhabited by algae, algae serve as an indication for determining the environmental safety^59^.

Previous research on the characterization and identification of *Streptomyces*-derived EA extract revealed the presence of twenty mixtures of organic compounds based on LCMS separation, whereas FTIR analysis confirmed the presence of fourteen functional groups^60^. Both Retnowati et al.^61^ and Ambarwati et al.^60^ reported similar results for functional groups isolated from *Streptomyces* EA extracts, including alkanes, alkyl, amines, aromatic amines, aromatic compounds, anhydrides, aryl, carboxylic acids, ether, imines/okime, phenols, secondary alcohols, 1.4 substituted/1,2,3,4 tetrasubstituted benzene and 1,3 substituted/1,2,4,4 substituted benzene. Paired with the advancements in bioinformatics technology, analyzing sequences from whole-genome sequencing using AntiSMASH software permits the prediction of chemical structures of compounds produced by *Streptomyces*. Although the production of SMs is strain-dependent, Ambarwati et al.^60^ proved that SMs such as SAL-2242 (lanthipeptide), albaflavenone (terpene), isorenieratene (terpene), geosmin (terpene), 7-prenilisatine (other), ectoine (other), melanin (other) and micromonolactam (PKS-1) showed 100% similarity to compounds recovered in other *Streptomyces* spp. Future research aims to identify genes involved in SM production and characterize the bioactive compounds synthesized by *Streptomyces* sp. KSF103 will benefit from the evaluation of their respective potentials in mosquito control.

Actinobacteria produce a vast range of bioactive compounds with a wide variety of biotechnology potentials, many of which have been applied for use in pharmaceuticals and as agricultural pesticides. However, as the number of documented metabolites expanded, it became clear that a growing number of previously unknown compounds were being rediscovered. As a result, it is imperative to identify novel compounds and explore uncharted microbial ecosystems. Future research involving the comprehensive identification and fractionation of bioactive compounds will pave the way for the compounds to be used in mosquito control.

## Conclusion

The present study reported that EA extracts produced by *Streptomyces* sp. KSF103 have insecticidal activities against various species of mosquitoes. Moreover, there is an absence of environmental toxicity in *Chlorella* spp. and *O. denticulata*. These findings indicate that the EA extract has significant potential for mosquito control and can further develop novel insecticidal formulations as an alternative to toxic chemicals for mosquito-borne diseases management. However, it should be kept in mind that successful vector control should not rely on merely an approach, but a plethora of innovations to fulfil different situations and end-users.

## Supplementary Information

Supplementary Information.

## Data Availability

All data generated or analyzed during this study are included in this article. Excel files can be provided on demand and should be addressed to Z.H.A.Y. The DNA sequence of *Streptomyces* sp. KSF103 is available in the GenBank repository, under the accession number MT355788 (https://www.ncbi.nlm.nih.gov/nuccore/MT355788.1/).
