# Supplementary material for: Insecticidal activities of Streptomyces sp. KSF103 ethyl acetate extract against medically important mosquitoes and non-target organisms
Source: Sci Rep. 2023 Jan 2;13:4. doi: 10.1038/s41598-022-25387-9 (PMC9807562; doi:10.1038/s41598-022-25387-9)
Supplement: Supplementary file 1 — Supplementary Information. [file 41598_2022_25387_MOESM1_ESM.docx]

1. **Supplementary Information**

Table S1. Biochemical profiling and photosynthetic performance between the treatment and control group for UMACC 313 *Chlorella* sp. and UMACC 258 *Chlorella* sp. (n = 3).

|  | **Chl-a** | **Carotenoid** | **Fv/Fm** | **Alpha (α)** | **rETRmax** | **Ek** |
| --- | --- | --- | --- | --- | --- | --- |
|  | **(mg/L)** | **(mg/L)** |  |  | **(µmol electrons m^-2^ s^-1^)** | **(µmol photons m^-2^ s^-1^)** |
| **UMACC 313 *Chlorella* sp. (Freshwater)** |  |  |  |  |  |  |
| *Streptomyces* sp. KSF103 EA extract |  |  |  |  |  |  |
| Day 0 | 1.084±0.097 | 0.450±0.053 | 0.375±0.057 | 0.263±0.063 | 65.669±11.981 | 251.935±26.747 |
| Day 4 | 4.390±0.108* | 1.879±0.036* | 0.483±0.044* | 0.350±0.031* | 147.397±30.850 | 417.778±51.273* |
| Day 8 | 4.377±0.301* | 2.030±0.089* | 0.411±0.046 | 0.282±0.026 | 78.100±14.690 | 275.715±25.827* |
| Day 12 | 5.031±1.152* | 2.352±0.461* | 0.388±0.027* | 0.243±0.011 | 78.670±17.043 | 322.714±56.238 |
| DMSO |  |  |  |  |  |  |
| Day 0 | 1.070±0.100 | 0.437±0.035 | 0.433±0.026 | 0.345±0.062 | 105.687±6.555 | 315.004±70.049 |
| Day 4 | 1.642±0.223 | 0.785±0.128 | 0.318±0.016 | 0.201±0.026 | 130.227±11.808 | 658.421±135.819 |
| Day 8 | 2.102±0.178 | 1.009±0.053 | 0.358±0.028 | 0.216±0.031 | 105.285±25.222 | 484.559±69.823 |
| Day 12 | 1.925±0.052 | 0.955±0.035 | 0.279±0.021 | 0.173±0.034 | 75.239±6.520 | 442.117±55.330 |
| **UMACC 258 *Chlorella* sp. (Marine)** |  |  |  |  |  |  |
| *Streptomyces* sp. KSF103 EA extract |  |  |  |  |  |  |
| Day 0 | 0.809±0.085 | 0.360±0.035 | 0.187±0.025 | 0.228±0.036 | 39.811±7.573 | 175.549±34.243 |
| Day 4 | 1.146±0.210 | 0.605±0.089 | 0.503±0.023* | 0.429±0.010* | 291.314±47.453* | 678.119±96.214* |
| Day 8 | 2.509±0.446 | 1.402±0.221 | 0.539±0.019 | 0.495±0.047 | 54.577±0.551 | 111.101±12.172 |
| Day 12 | 3.853±0.397* | 2.285±0.664* | 0.557±0.040 | 0.513±0.081* | 50.817±2.362 | 100.329±12.806 |
| DMSO |  |  |  |  |  |  |
| Day 0 | 0.801±0.071 | 0.368±0.038 | 0.227±0.030 | 0.274±0.017 | 39.240±1.362 | 143.282±7.205 |
| Day 4 | 1.138±0.164 | 0.630±0.064 | 0.413±0.030 | 0.275±0.064 | 678.119±96.214 | 307.065±14.891 |
| Day 8 | 2.074±0.108 | 1.019±0.054 | 0.500±0.040 | 0.483±0.024 | 36.568±1.231 | 75.894±5.868 |
| Day 12 | 1.914±0.338 | 1.004±0.094 | 0.517±0.054 | 0.309±0.017 | 36.313±3.847 | 118.309±18.329 |

* significant at P < 0.05 level versus negative control. Data expressed as mean±standard deviation. Maximum quantum efficiency (Fv/Fm) is a parameter to indicate the physiological state of phytoplankton. Alpha is photosynthetic efficiency, and it indicates the amount of ETR per photon. Ek is the photoadaptive index and it indicates how well cells are adapted to their light environment
